# Co-creating point-of-care ultrasound education for family medicine in Ethiopia: reflections on pedagogy, faculty development, and academic partnership

**DOI:** 10.3389/fmed.2026.1926736

**Published:** 2026-10-02

**Authors:** Bilal Negash, Leila Makhani, Jamie Rodas, Eden Habtemariam, Praseedha Janakiram, Michael Bartucci

**Affiliations:** 1 Temerty Faculty of Medicine, University of Toronto, Toronto, ON, Canada; 2 Addis Ababa University School of Medicine, Addis Ababa, Ethiopia

**Keywords:** academic partnership, co-creation, collaboration, Ethiopia, faculty development, family medicine, pedagogy, point—of—care diagnostics

## Abstract

Point-of-care ultrasound (POCUS) is increasingly becoming an essential clinical and educational tool in low to middle income countries (LMICs), where formal imaging is often delayed or inaccessible. This commentary reflects on the co-creation and longitudinal implementation of a Family Medicine-focused point-of-care ultrasound education initiative at Addis Ababa University (AAU), developed through the Toronto Addis Ababa Academic Collaboration in Family Medicine (TAAAC-FM). We discuss how this initiative, while addressing an immediate diagnostic need, also functions as a curricular lever for faculty development, a pathway for professional identity formation, and can foster unique pathways for ongoing departmental sustainability. We discuss the partnership structures and equity considerations that shaped program design and situate this work within broader efforts to strengthen Primary Health Care (PHC) and advance Universal Health Coverage (UHC). These reflections offer transferable insight for academic collaborations seeking to align educational innovation with health system transformation in resource-constrained settings.

## Introduction: background and rationale

TAAAC-FM is a long-standing partnership between the Department of Family and Community Medicine (DFCM) at the University of Toronto (U of T) and the Family Medicine Unit (FMU) at AAU (1). Since 2013, when Family Medicine was first established as a specialty in Ethiopia, the TAAAC-FM partnership has focused on using clinical education to empower local faculty by building sustainable institutional growth within AAU (2). This approach reflects the broader TAAAC model, which emphasizes growth through reciprocal knowledge exchange and local ownership. Rather than a unidirectional transfer of knowledge from a higher-resource to a lower-resource institution, this model prioritizes educational co-creation through developing curricula that best reflects realism and sustainability long-term (3). With this, the intended outcome is to help support the development of faculty who are now increasingly positioned to teach, lead and build even more educational capacity within their institution and health system.

POCUS education has emerged as a particularly relevant focus within the TAAAC-FM partnership. In Ethiopia, as in many LMICs, access to formal imaging such as timely ultrasound, CT, or MRI, is often limited and contributes to prolonged diagnostic uncertainty (4). Patients frequently present later in their disease course, reflecting broader barriers to health service access in their healthcare system (5). In this context, POCUS allows clinicians to obtain actionable bedside diagnostic information without the delays associated with formal imaging pathways (6, 7). Its portability and durability make it especially well suited to lower-infrastructure settings, and its applications span a wide range of clinical domains relevant to Family Medicine, including assessment of fluid volume status, evaluation of dyspnea, evaluation of abdominal pathology, obstetrical screening, and procedural guidance (8). With this in mind, a challenge to POCUS utilization by local clinicians in Ethiopia seems tied to a lack of an organized curriculum and lack of available machines (8). To date, there is limited to no data that has focused on POCUS utilization following a formal educational curriculum and the impacts this could have at a regional level. In response to a need voiced directly by AAU FMU faculty for structured POCUS training, DFCM faculty and the AAU FMU collaborated through the TAAAC-FM partnership to co-create a novel POCUS training pathway for AAU FMU faculty, designed to be both clinically rigorous and locally sustainable.

This work also responds directly to broader health system priorities. Strengthening PHC is central to achieving UHC and the health-related Sustainable Development Goals (SDGs), particularly SDG 3 (good health and well-being). Diagnostic capacity at the point of first contact is a recognized determinant of whether health systems that follow PHC principles can deliver timely, equitable care. By embedding diagnostic skill-building directly within Family Medicine, a discipline positioned at the front line of primary care in Ethiopia, this initiative contributes to the broader project of PHC transformation. It strengthens the clinical competence of the workforce most likely to encounter undifferentiated illness early and empowers them to make actionable clinical decisions in real-time for patients across the lifespan.

## Pedagogical framework and underlying competencies

The TAAAC-FM POCUS education and training program is built primarily on the existing Canadian Point of Care Ultrasound Society (CPOCUS) certification framework, delivered through an established CPOCUS training bootcamp held in Nairobi, Kenya, and Dar es Salaam, Tanzania by an organization called POCUS Outreach. This training pathway leads to Independent Practitioner (IP) certification in the acute core POCUS indications, namely the abdominal aorta exam, abdominal free fluid exam, subxiphoid cardiac exam, pleural effusion exam, pneumothorax exam, and first-trimester obstetrics exam. In addition to these competencies, there is modular certification in the inferior vena cava exam, respiratory distress lung protocol, and comprehensive lung exam. Upon completion of the acute core POCUS certification and the modular certifications above, participants obtain a unique certification tier developed for this collaboration, referred to as Acute Core Plus.

CPOCUS is a nationally recognized Canadian certification body, and its standards provide the AAU FMU faculty with a level of training proficiency that is rigorous and externally validated. Importantly, this certification model has since been adopted more broadly across East Africa, suggesting that the framework co-developed through this partnership may have relevance well beyond the immediate AAU context. AAU FM faculty who complete certification are encouraged to apply these skills directly in their clinical duties and in the teaching of Family Medicine residents, positioning certified faculty as the foundation for internal teaching capacity rather than as recipients of a one-time external service.

Supplementing the rigorous POCUS certification experienced in Kenya or Tanzania, the AAU FMU faculty were provided with single-day combination didactic/hands-on POCUS teaching sessions twice per year by visiting DFCM faculty, and hands-on POCUS utilization mentorship by visiting DFCM faculty who had POCUS certifications matching the CPOCUS certified AAU FMU faculty. The single-day teaching sessions were provided to all AAU FMU faculty as well as to all Family Medicine residents, and served multiple purposes including: refreshing the POCUS skillset of AAU faculty who already completed POCUS certification; introducing the POCUS skillset to AAU faculty who had not completed POCUS certification; and introducing the POCUS skillset to the AAU Family Medicine residents.

Two handheld POCUS devices were procured for use solely by AAU FM faculty, a deliberate choice intended to protect equipment access for those who had invested in training and to support consistent clinical integration of the skill. These POCUS devices were chosen based on multiple criteria, including: ability to be used without wifi, long operation times without access to chargers, quality of images generated, and versatility of each probe in image generation/exam capacity. These devices were also selected from the primary devices utilized to teach the POCUS training program in Kenya/Tanzania, allowing for immediate familiarity and proficiency with these devices upon return from the certification program.

## Co-creation and partnership process

A defining feature of this initiative is that it was not designed externally and delivered to the AAU FMU faculty, but rather was built jointly in response to a need that the AAU FMU faculty themselves identified. The decision of which POCUS competencies to prioritize and how training would be structured emerged from sustained dialogue between DFCM and FMU faculty.

This co-creation extended to decisions about feasibility and pacing. Faculty selection for training cohorts was made by the sitting AAU FMU program director based on nomination and physician availability, keeping the selection process embedded within local departmental leadership rather than dictated by the visiting partner. Site selection for the in-person bootcamp and the decision to pursue a regionally available certification pathway, rather than requiring travel to North America, were similarly shaped by what AAU faculty identified as feasible given clinical workload and travel constraints.

This approach reflects a broader orientation within TAAAC-FM toward reciprocal rather than unidirectional partnership. AAU faculty are not positioned solely as trainees but as co-designers of the curriculum and, increasingly, as the eventual teaching faculty for their own residents. This shift, from external delivery toward internal ownership, is intended to mitigate the power asymmetries that can characterize global health education partnerships, and to support the kind of institutional embedding that has been associated with program durability in comparable LMIC POCUS initiatives (9).

## Learning environment, objectives, and pedagogical format

Training took place over a 5 day bootcamp and focused on Acute Core Plus POCUS indications, held in either Nairobi or Dar es Salaam. Participants completed an online didactic curriculum that must be passed prior to attending the in-person component. The majority of in-person bootcamp time is spent scanning ultrasound models to obtain the threshold number of determinate scans required for certification in each individual POCUS application.

The learning objectives guiding this work are to: successfully train and certify participating faculty in the Acute Core Plus indications; translate certified skills into routine clinical use at AAU; establish an AAU FM resident POCUS teaching program led by certified AAU faculty themselves; and, longer term, contribute to the development of broader East African POCUS training networks through this cross-border model.

## Results to date

Of the six AAU FM faculty trained through this program, four have achieved full Acute Core Plus certification, and two have achieved Acute Core certification. Faculty have begun incorporating these skills into inpatient and outpatient care, including using POCUS to support earlier identification of conditions such as pleural effusions in metastatic cancer patients, allowing for safer and earlier diagnostic and therapeutic thoracenteses when alternative imaging can otherwise take several more days or weeks to complete. Similar applications have been observed in assessment of fluid volume status, other respiratory pathology, and obstetric care, consistent with findings from the broader Ethiopian and LMIC POCUS literature (4, 8).

A qualitative study examining the facilitators and barriers to the adoption of POCUS training and skills within the AAU FMU clinical environment is currently underway, with research ethics recently approved by both U of T and AAU. This evaluation is intended to capture not only clinical uptake but the lived experience of faculty navigating their new diagnostic capability within existing resource and workflow constraints. This data will directly inform future iterations of this co-created training program.

## Equity considerations

Equity has shaped this initiative at several levels. At the level of patient care, POCUS has the potential to narrow diagnostic disparities between patients who can access formal imaging in a timely fashion and those who cannot, a gap that disproportionately affects patients presenting with acute or advanced disease in resource-constrained settings. Embedding POCUS within Family Medicine, rather than restricting it to emergency or subspecialty contexts, extends this diagnostic benefit across both acute and longitudinal primary care encounters.

At the level of the partnership itself, curricular and logistical decisions rest with AAU leadership in collaboration with TAAAC-FM, and the long-term goal of the collaboration is local Ethiopian faculty-led teaching rather than ongoing dependence on external trainers. This is consistent with recognized principles for equitable global health partnerships, in which sustainability is judged not by the duration of external involvement but by the speed with which that involvement becomes unnecessary (9).

## Discussion: practical implications and lessons learned

This initiative illustrates how a single clinical skill, deliberately co-created and longitudinally supported, can serve multiple functions simultaneously. POCUS strengthens bedside diagnostic capacity in a setting where formal imaging is frequently delayed or inaccessible, while also functioning as a structured vehicle for faculty development and departmental identity formation within a Family Medicine program that remains administratively embedded within internal medicine and is still establishing its institutional footprint. Previous work and research in this area is still limited and mostly focused on emergency and critical care settings rather than a family medicine faculty development context. Similarly, these studies have identified structured training, equipment access and continuous opportunities for practice important determinants of POCUS uptake (8–10).

Several lessons have emerged from this training experience. Recognized external certification, such as that offered through CPOCUS, appears to enhance both credibility and motivation among faculty, while still allowing flexibility in how skills are applied to local clinical needs. This is consistent with prior Ethiopian POCUS initiatives demonstrating that the initial training alone may improve knowledge and confidence, but that ongoing mentorship and locally embedded teaching structures that could allow for more opportunities to practice are important for translating training into sustainable usage (9, 10). Our initiative differs in its emphasis on faculty certification and the eventual development of faculty as local POCUS champions and educators. A defined POCUS competency also offers Family Medicine a distinctive clinical and educational niche in an academic environment where departmental visibility can otherwise be limited. Critically, this model depends on continuous mentorship and repeated clinical integration; isolated training events, without follow-up and clinical reinforcement, are unlikely to produce sustained competency (10). Faculty engagement has been highest when curriculum content and pacing are co-created and aligned with local clinical realities rather than imposed as a fixed external standard. There is also an early but promising cascade effect: equipping a small number of faculty with certified skills appears to be extending POCUS exposure to residents and other learners, which may, over time, broaden confidence and capacity well beyond the initially trained cohort (7, 9).

For PHC transformation specifically, this experience suggests that diagnostic skill-building at the primary care level can be a meaningful lever for system strengthening, not only because it improves point-of-care decision making, but because it builds workforce capacity and confidence and may create ongoing opportunities for advanced clinical and academic development within local systems. This points to a broader lesson for UHC and SDG goals: training that is locally led and built to last in turn keeps delivering clinical and educational benefit long after a visit ends, unlike a short-term clinical service trip where most of the benefit leaves in tandem with the departure of the visiting team.

## Constraints and limitations

The bootcamp-style, intensive training format also carries trade-offs. Concentrated in-person training allows rapid skill acquisition and certification but requires faculty to be away from clinical and teaching duties at AAU for an extended period, and depends on continued access to a small number of shared devices to consolidate skills afterward. These trade-offs will likely inform how future iterations of this training are structured, including whether elements of the model can eventually be delivered locally at AAU rather than requiring regional travel.

The limited number of available POCUS devices restricts both the scalability of clinical use and the ability to extend hands-on resident teaching and may contribute to skill decay among certified faculty if scanning opportunities remain infrequent between training visits. Formal outcome data linking POCUS use to measurable changes in patient clinical outcomes has not yet been collected and represents an important next step for evaluating the program's clinical impact beyond faculty certification numbers.

The findings described throughout this paper should also be interpreted within the context of the early stage of this initiative. The number of formally trained faculty remains small, and certification status does not equate to long-term retention, frequency of clinical use, teaching effectiveness, or better impact on clinical decision making. For this, formal pre- and post- training measures should be conducted to assess scanning competency and utilization clinically. As such, the observations presented here should be interpreted primarily as an educational experience of early implementation rather than evidence of program effectiveness.

As well, broader integration of POCUS into the AAU Family Medicine residency curriculum will require dedicated protected teaching time, which may not yet be feasible while faculty continue to consolidate their own certification and while device availability remains limited. Future evaluation should examine outcomes at several levels, including faculty competency, frequency, breadth of use for POCUS clinically, assessing progression to independent teaching roles and resident uptake. Also, clinical outcomes such as changes in patient outcomes, diagnostic pathways, formal imaging utilization and referrals will also be important to assess.

## Conclusion

The integration of POCUS into Family Medicine education at AAU demonstrates how curriculum, instruction, and pedagogy can be deliberately aligned with both clinical necessity and institutional context. In a setting where diagnostic delay and advanced disease burden remain persistent challenges, POCUS functions as a competency-based educational tool capable of empowering local clinicians while simultaneously strengthening patient care, teaching capacity, empowering faculty and shaping departmental identity. When built through co-creation and sustained faculty development, this kind of educational innovation can offer a transferable model for academic collaborations seeking to strengthen primary care education and advance health system transformation in resource-constrained settings.

## Data Availability

The original contributions presented in the study are included in the article/Supplementary Material, further inquiries can be directed to the corresponding author.
